# Potential risk zone for anthropogenic mortality of carnivores in Gandaki Province, Nepal

**DOI:** 10.1002/ece3.8491

**Published:** 2022-01-12

**Authors:** Binaya Adhikari, Kedar Baral, Shivish Bhandari, Michelle Szydlowski, Ripu M. Kunwar, Saroj Panthi, Bijaya Neupane, Raj Kumar Koirala

**Affiliations:** ^1^ Institute of Forestry Tribhuvan University Pokhara Nepal; ^2^ Pokhara Zoological Park & Wildlife Rescue Center Kaski Nepal; ^3^ Division Forest Office Kaski Nepal; ^4^ School of Natural and Computational Science Massey University Auckland New Zealand; ^5^ Morgan State University Baltimore Maryland USA; ^6^ Department of Anthrozoology University of Exeter Exeter UK; ^7^ Food and Agricultural Organization Kathmandu Nepal; ^8^ Ministry of Forest, Environment and Soil Conservation Pokhara Nepal

**Keywords:** casualty, conflict, death, human, leopard, wildlife

## Abstract

Anthropogenic pressures in human‐dominated landscapes often contribute to wildlife mortality. Carnivores are especially vulnerable to human‐induced mortality due to the perceived threat to livestock and humans. Despite having widespread conservation implications, carnivore mortality data have been largely underutilized within Nepal. This study utilized Maxent to identify high‐risk areas and explore the contribution of habitat attributes associated with carnivore mortality using the casualty database within the Gandaki province of central Nepal. We categorized the risk to carnivore species in three taxonomic groups, Felid, Viverridae, and Herpestidae, and identified a 3704‐km^2^ area within the province at high risk for carnivore casualty. The middle mountains were the riskiest physiographic zone, and the Annapurna Conservation Area represented the largest risk zone among the four protected areas. Agricultural land was the most problematic area in terms of carnivore casualty. The human population was positively associated with high‐risk areas and the number of casualties, whereas protected area cover had a negative association. This study identified that the common leopard was at the highest risk of mortality and therefore would benefit from the implementation of an action plan and species‐specific conservation strategies, especially within identified high‐risk zones. An expansion of protected areas in the middle mountain region would serve to greatly reduce carnivore casualty. Species distribution modeling can be further used with national‐level spatial and temporal mortality data to identify the most prominent casualty times and pinpoint potential casualty locations throughout the country.

## Graphical abstract

In this study, maximum entropy model was used to identify high‐risk areas and explore the contribution of habitat attributes associated with carnivore mortality using the casualty database within the Gandaki province of central Nepal. We identified a 3704‐km^2^ area within the province at high risk for carnivore casualty. This study identified that the common leopard was at the highest risk of mortality and therefore would benefit from the implementation of an action plan and species‐specific conservation strategies, especially within identified high‐risk zones.

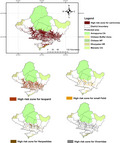

## 1 INTRODUCTION

Human–wildlife conflict (HWC) often arises in shared landscapes, where human settlements overlap with wildlife habitat, impacting multiple species (Batavia & Nelson, 2016; Madden, 2004). HWC refers to the negative impacts generated by the interaction of wildlife and humans and creates numerous challenges for the conservation of threatened species around the world (Dickman, 2010; Distefano, 2005). These negative impacts can refer to the loss of human life, property, or the loss of wild species and their habitat or resources. One of the gravest consequences of HWC is the unnatural mortality of wildlife via anthropogenic causes (Cline et al., 2007; Hill et al., 2020). More than a quarter of global terrestrial vertebrate mortalities are associated with human activities (Hill et al., 2019), which can act as a barrier for survival if viable breeding adult populations are lost (Bennett et al., 2009; Fuller, 1989; Mclellan et al., 1999), and might result in the extinction of various species of wildlife (Frankham, 2005; Shaffer, 1981). Among highly impacted species, larger mammals are more vulnerable to human‐induced mortality compared with smaller species (Hill et al., 2020). Carnivore species are often perceived as threats and are frequently associated with conservation discourses due to their predatory habits on livestock, pets, and even humans (Chapron & Treves, 2016; Kuijper et al., 2016; Loveridge et al., 2017). Incidences of carnivore mortality have increased in recent years, with human‐induced mortality being the most prominent threat to their survival (Parchizadeh & Belant, 2021; Taylor‐Brown et al., 2019). Illegal hunting (Pohja‐Mykrä, 2016), prey poisoning (Bhandari & Chalise, 2016; Mateo‐Tomas et al., 2012), and road mortality (Seiler & Helldin, 2006) are the primary causes of human‐induced carnivore mortality globally (Inskip et al., 2014; Kissui, 2008; Merson et al., 2019; Swanepoel et al., 2014). Nepal is not exempt from these anthropogenic pressures (Bhandari & Chalise, 2016; Bhandari et al., 2019; Dinerstein & Meheta, 1989; Thapa, 2014).

Understanding the attributes of mortality and the severity of risk in specific areas can be vital to conservation endeavors, especially in areas of high HWC. For example, studies regarding the circumstances of mortality are an important consideration in understanding population dynamics and the risks associated with the loss of breeding pairs (Bekoff, 2001; Mateo‐Tomas et al., 2012; Seiler & Helldin, 2006; Taylor‐Brown et al., 2019). The long‐term survival of adults, especially females, is crucial for maintaining a viable population of carnivores (Knight & Eberhardt, 1985; Purvis et al., 2000; Weaver et al., 1996). Mortality data can be utilized to determine the sustainability of a given population and can provide insights on the methods needed to minimize overall mortality rates (Goodrich et al., 2008).

To better understand HWC and the spatiotemporal interactions between humans and wildlife, the species distribution model (SDM) has been employed to map areas of importance to carnivore survival (Kalle et al., 2013; Kramer‐Schadt et al., 2013). The usage of SDM in the field of predicting risk for wildlife can help conservation endeavors by narrowing the focus to include only the economic and management efforts which are required to develop conservation plans for specific sites within a given timeframe (Mateo‐Tomás et al., 2012). Maxent is a widely used approach for SDM (Fitzpatrick et al., 2013; Phillips et al., 2006) and utilizes sample background location data contrasted against presence location data to estimate potential occurrence zones (Phillips et al., 2006). It has demonstrated the best predictive power across all sample sizes and has shown superior performance compared with other modeling software even when sample sizes are low (Elith et al., 2011; Wisz et al., 2008). This approach has previously been used in various studies to map suitable habitats for wildlife under the influences of anthropogenic, environmental, and topographic variables (Bai et al., 2018; Panthi et al., 2019; Sharma et al., 2020; Thapa et al., 2018). Though Maxent has been used to assess and map risk areas as well as predict mortality risk zones for various wildlife around the world (Garrote et al., 2018; Mateo‐Tomás et al., 2012), its application to carnivores in Nepal is novel.

In Nepal, most of the studies on the HWC have focused on risks to human lives, livestock, crops, and infrastructure (Acharya et al., 2016; Aryal et al., 2014; Koirala et al., 2012; Sapkota et al., 2014; Sharma et al., 2021). However, dimensions of risk and casualty to wildlife have been largely understudied. This study, therefore, aimed to map potential risk zones for carnivore mortality using SDM. We aimed to identify a potential risk zone for four carnivore groups and analyze the attributes associated with their mortality. We hypothesized that high‐risk areas for these carnivores are associated with agricultural lands and anthropogenic structures (e.g., road, settlements) in non‐protected regions. We also hypothesized that the risk area for the common leopard (*Panthera pardus*) is greater than the risk to smaller carnivores.

## 2 MATERIALS AND METHODS

### 2.1 Study area

**FIGURE 1:**
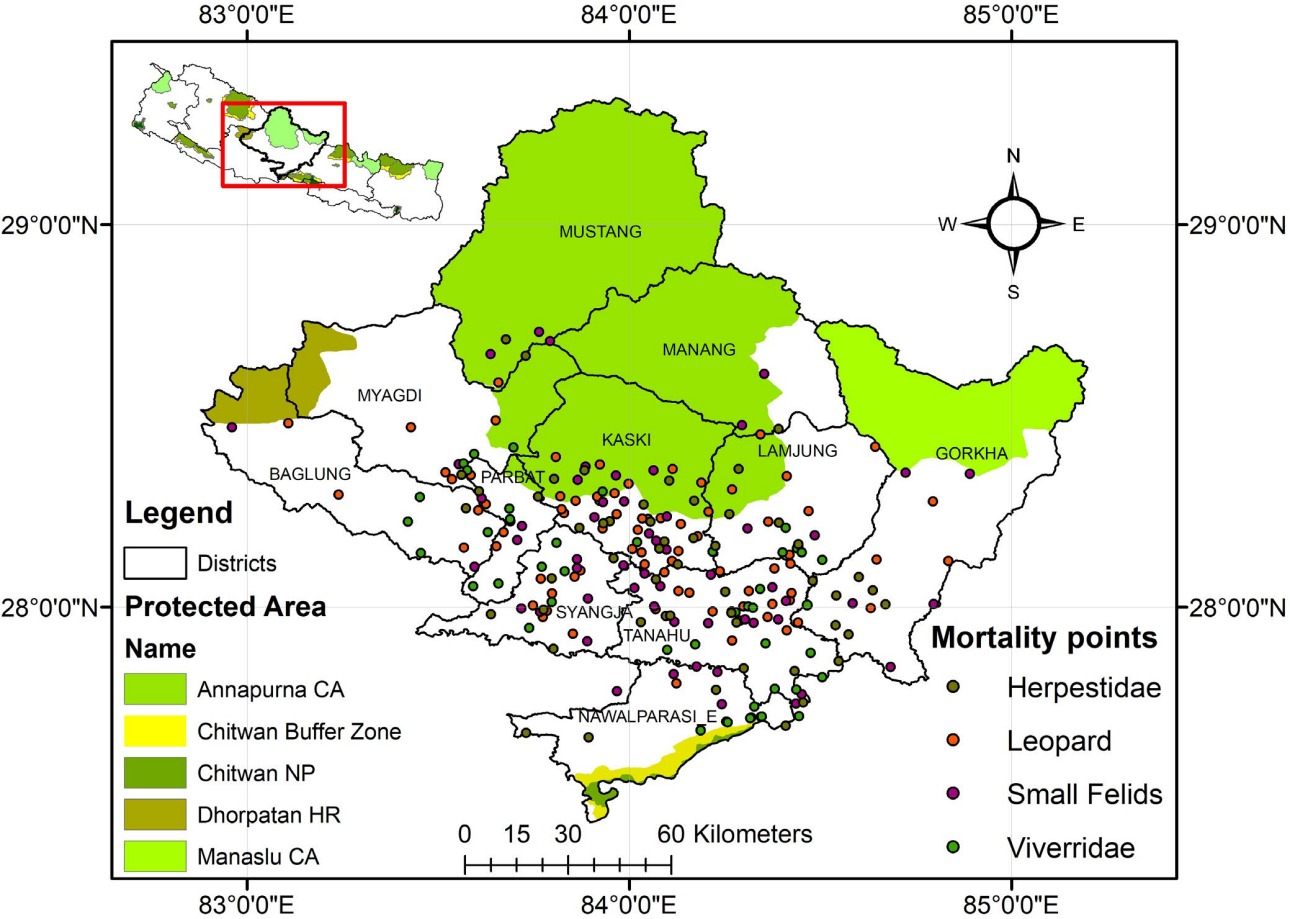
Map of study area representing 11 districts within province, four protected areas and mortality locations of Herpestidae, Leopard, small felid, and Viverridae

The study area lies within the Gandaki province (Figure 1) with a total area of 21773 km^2^ (14.6% of Nepal's area) and includes five physiographic zones (High Himalayas, High mountains, Middle mountain, Siwalik, and Terai) (Table 1) and 11 administrative districts (Baglung, Gorkha, Kaski, Lamjung, Manang, Mustang, Myagdi, Nawalpur, Parbat, Syangja, and Tanahun). The total population of the Gandaki province is 2,403,041 (9.06% of Nepal's total population), and the majority of people (72.4%) are involved in agriculture such as farming and livestock. Main livestock species in the province include cow (*Bos taurus*), buffalo (*Bubalus bubalis*), domestic goat (*Capra aegagrus hircus)*, and domestic sheep (*Ovis aries*). Other common domestic animals include dogs (*Canis lupus*) and cats (*Felis silvestris catus*). Many local people rely on forest provisioning for subsistence, including the use of firewood, fodder, and medicinal plants (Baral et al., 2019; Kutal et al., 2021).

**TABLE 1:** The description of the five physiographic regions in Nepal mentioning altitude, climate, and vegetation type

Physiographic regions	Characters
Terai	(below 500) m.a.s.l, covers, tropical and sub‐tropical climate, major forest: *Shorea robusta*, *Acacia catechu*, etc
Siwalik	(500–1000) m.a.s.l. sub‐tropical climate, major forest: *Shorea robusta*, *Acacia catechu*, *Alnus nepalensis*, etc
Middle mountain	(1000–3000) m.a.s.l. sub‐tropical climate at the bottom of the hills but gradually cooler, major forest: *Alnus nepalensis*; *Castanopsis* spp., *Rhododendron* spp
High mountain	3000 to 5000 m.a.s.l. cold temperate climate, major forest: *Pinus* spp., *Rhododendron* spp
High Himalayas	above 5000 m.a.s.l. alpine to tundra climate, *Juniperus*‐*Rhododendron* association, alpine scrub

The Gandaki province includes four protected areas (Annapurna Conservation Area (CA), Manaslu CA, Dhorpatan Hunting Reserve (HR), and Chitwan National Park (NP)). The study area has forest coverage equaling 7138.3 km^2^ (32.6%) which is home to more than 55 mammalian species (Baral et al., 2019). Some of the major carnivore species found in the province are felid species (common leopard (*Panthera pardus*), tiger (*Panthera tigris*), snow leopard (*Panthera uncia*), clouded leopard (*Neofelis nebulosa*), jungle cat (*Felis chaus*), and leopard cat (*Prionailurus bengalensis*)). Representing Viverridae are the large Indian civet (*Viverra zibetha*), common palm civet (*Paradoxurus hermaphrodites*), and masked palm civet (*Pagumalarvata*). Herpestidae includes Indian gray mongoose (*Herpestes edwardsii*), the small Indian mongoose (*Herpestes auropunctatus*), and the crab‐eating mongoose (*Herpestes urva*). Representing Canidae is the golden jackal (*Canis aureus*), red fox (*Vulpes vulpes*), and Himalayan wolf (*Canis lupus*), and Ursidae includes the Himalayan black bear (*Ursus thibetanus*) and brown bear (*Ursus arctos*). Ailuridae such as the Red panda (*Ailurus fulgens*), Mustelidae such as the Eurasian otter (*Lutra lutra*), and yellow‐throated marten (*Martes flavigula*) are also found in the area (Baral et al., 2019).

### 2.2 Data collection

The study area was divided into eleven blocks in accordance with administrative districts. The study blocks represented five physiographic zones and included five protected areas of the province, hereby capturing the heterogeneity of physiography and protected/non‐protected regions. The division forest offices of each district, wildlife rescue center, and the protected area offices were visited for secondary data collection regarding wildlife casualties between January 2019 and February 2021. Information on incident attributes such as the name of affected species, location of mortality (GPS coordinates), cause of mortality, age of individual, and season of mortality was recorded. The casualty location specified in the database of the divisional forest, protected areas (PA), and wildlife rescue center was verified through field visit and key informant interviews. The cause of mortality was further grouped in 4 categories: retaliation killing (poisoning, gunshot, injured by sticks, stones, or sharp objects), roadkill (vehicle collision), injured by feral dogs (indication of dog bite marks), and other unknown causes.

A total of 306 cases of mortality (from 9 species within three taxonomic family of carnivore) were identified throughout the study period. Out of the total cases, the cases with insufficient information regarding location, species, age, or cause of mortality were omitted, hereby retaining only 232 mortality points for further analysis. Those 232 points were further categorized into three groups of taxonomic family (felid, Viverridae, and Herpestidae) for this study due to their high casualty frequencies inside the province compared with other carnivore families. The felid family included three species: the common leopard, leopard cat, and jungle cat. We further categorized felid into two subgroups: large felid (common leopard) (*N* = 64) and small felid (*N* = 56) (leopard cat, jungle cat). The Viverridae family (*N* = 60) included the large Indian civet, common palm civet, and masked palm civet. The Herpestidae family (*N* = 52) included the Indian gray mongoose, the small Indian mongoose, and the crab‐eating mongoose.

### 2.3 Selection of variables for modeling

We collected information on topographic, vegetation, and anthropogenic variables (Table 2) from several sources to use in maximum entropy modeling. The environmental variables considered in our models have been previously utilized for identification of suitable habitat (Sharma et al., 2020) and risk zone mapping (Karki & Panthi, 2021).

**TABLE 2:** Initial variables used in VIF test stepwise elimination process to be further used in Maxent modeling. Table represents sources of data used in the study, category, and unit of those variables

Source	Category	Variable	Unit	Web sites
USGS	Topographic	Elevation	m	https://earthexplorer.usgs.gov/
		Aspect	Degree	
		Slope	Degree	
GEOFABRIK		Distance to water	m	https://www.geofabrik.de/data/shapefiles.html
Landsat	Vegetation‐related	Mean EVI, Min EVI, Max EVI	Dimensionless	(https://earthexplorer.usgs.gov/)
GFC		Forest	Dimensionless	Global Forest Change (GFC) Web site Hansen et al. (2013)
GEOFABRIK	Anthropogenic	Distance to settlement	m	https://www.geofabrik.de/data/shapefiles.html
		Distance to motor road	m	
		Distance to path	m	
		Distance to building	m	
HUMDATA		Population density	Dimensionless	https://data.humdata.org/
ICIMOD		Land use/land cover	m	http://www.icimod.org) Uddin et al. (2015)

Digital elevation model (DEM) having 30‐m resolution was downloaded from the Web site of United States Geological Survey (https://earthexplorer.usgs.gov/). Slope and aspect were derived from the DEM using ArcGis software (ESRI, 2017). Shape files of water sources were downloaded from the Geofabrik Web site (https://www.geofabrik.de/data/shapefiles.html) and converted to distance raster file using ArcGIS (ESRI, 2017). Since the climatic variables were not available in 30‐m resolution, elevation was taken as a proxy of temperature. Data associated with forest cover were downloaded from the Global Forest Change (GFC) Web site (Hansen et al., 2013). The Enhanced Vegetation Index (EVI) time‐series data of 2018 and 2019 which were derived from images of Landsat 8 were analyzed with the help of the Google Earth Engine.

The anthropogenic variables were also used in our models. The shape files available on the Geofabrik Web site (https://www.geofabrik.de/data/shapefiles.html) were used for information on the location of paths and roads. The information on locations of settlements was derived from the Department of Survey, Nepal. ArcGIS was utilized to create distance raster files of paths, roads, and settlements (ESRI, 2017). Land‐use and land cover (LULC) data were downloaded from the International Centre for Integrated Mountain Development Web site (ICIMOD; http://www.icimod.org) (Uddin et al., 2015). Information on population density was obtained from humdata Web site (https://data.humdata.org/) and processed in ArcGIS. All variables were pre‐processed in ArcGIS (ESRI, 2017) to convert in ASCII format and final spatial resolution of 30 m.

### 2.4 Data analysis

The descriptive mortality data regarding the family, cause, age, and season were analyzed for the significant differences and association between the variables using chi‐squared test of homogeneity and goodness of fit. The study focused on maintaining accuracy on prediction of high‐risk zones for various groups of carnivores. Efficiency of model prediction is improved by accounting uneven sampling bias (Kramer‐Schadt et al., 2013), and hence, spatial filtering in oversampled region was deemed useful (Phillips et al., 2009). The occurrence of mortality was therefore filtered by maintaining at least 100 m of distance between the points within each group of carnivores, hereby minimizing spatial autocorrelation (Karki & Panthi, 2021; Sharma et al., 2020). 58 points out of 64 leopard mortality points, 50 points out of 56 small felid mortality points, 53 points out of 60 Viverridae mortality points, and 50 out of 52 Herpestidae mortality points were retained for modeling after removing spatially autocorrelated points. Variance inflation factor (VIF) test was conducted using R script (R Core Team, 2020) to reduce multicollinearity by omitting highly correlated variables with VIF > 3 (Zurr et al., 2010). The variables (Table 2) were removed by stepwise elimination process until only the variables with VIF < 3 remained. This process was repeated with variables for all four groups (leopard, small felid, Viverridae, and Herpestidae) of carnivores, and the remaining variables were utilized to generate models. Maxent software (version 3.4.4) was configured to utilize 70% of the casualty points for training the model and 30% for model validation (Karki & Panthi, 2021). A limit of 1000 maximum iterations was selected, and models were replicated ten times for each carnivore group to generate average model information (Barbet‐Massin et al., 2012). Accuracy of the models was assessed with both threshold‐dependent and threshold‐independent methods. In the threshold‐independent model, the area under receiver operator curve (AUC) value was obtained to evaluate model performance. A value < 0.7 represented poor model performance, 0.7–0.9 represented moderate performance, and a value greater than 0.9 represented excellent performance (Pearce & Ferrier, 2000). Even though AUC is widely used model evaluation parameter, it is severely criticized by researchers (Lobo et al., 2008). To overcome this limitation, it is recommended to use AUC in combination with methods such as AIC (Akaike information criterion) or TSS (True skill statistics). Using R software (R Core Team, 2020), we used threshold‐dependent method, TSS (sensitivity + specificity −1) which places equal weight on model sensitivity and specificity, with its value ranging from −1 to 1 (Allouche et al., 2006).

The threshold of maximum sum of sensitivity and specificity is recommended to generate information on distribution using presence‐only data (Liu et al., 2013). This threshold was used to convert the continuous probability map into a binary map consisting of high‐ and low‐risk probabilities. Areas with probability values above the threshold were categorized as high‐risk zone whereas the areas with lower probability value than the threshold were categorized as low‐risk zone. The high‐risk zones obtained for each group of carnivores were not mutually exclusive and could overlap with each other; therefore, the overall carnivore risk zone was obtained by creating union of all four high‐risk zone layers of carnivore groups in ArcGIS. The high‐risk shapefile was then intersected over land use/land cover, district, protected areas, and physiographic zones within the province to obtain the intersected data.

We performed Canonical Correspondence Analysis (CCA) (TerBraak & Smilauer, 2002), which is a multivariate constrained ordination technique. This method extracts major gradients among combinations of explanatory variables in a dataset. We selected this method because we had categorical district wise data and the independent variables were consistent within the sample site.

CCA was utilized to determine the association of high‐risk area (in km2) within each district and the number of casualty in each district, with several environmental and anthropogenic variables (forest cover, livestock holding, human population, protected area cover) within each district. These variables are frequently mentioned in literatures (Distefano, 2005; Manral et al., 2016; Peterson et al., 2010; Sangay & Vernes, 2008) influencing the intensity of human–carnivore conflict. The data regarding high‐risk area (km^2^) for each district were extracted from Maxent (union of high‐risk areas of four groups of carnivores) using ArcGIS, whereas district wise data on other variables such as forest cover, livestock holding, and human population were extracted from opendatanepal Web site (https://opendatanepal.com/).

## 3 RESULTS

### 3.1 Statistics of mortality

**FIGURE 2:**
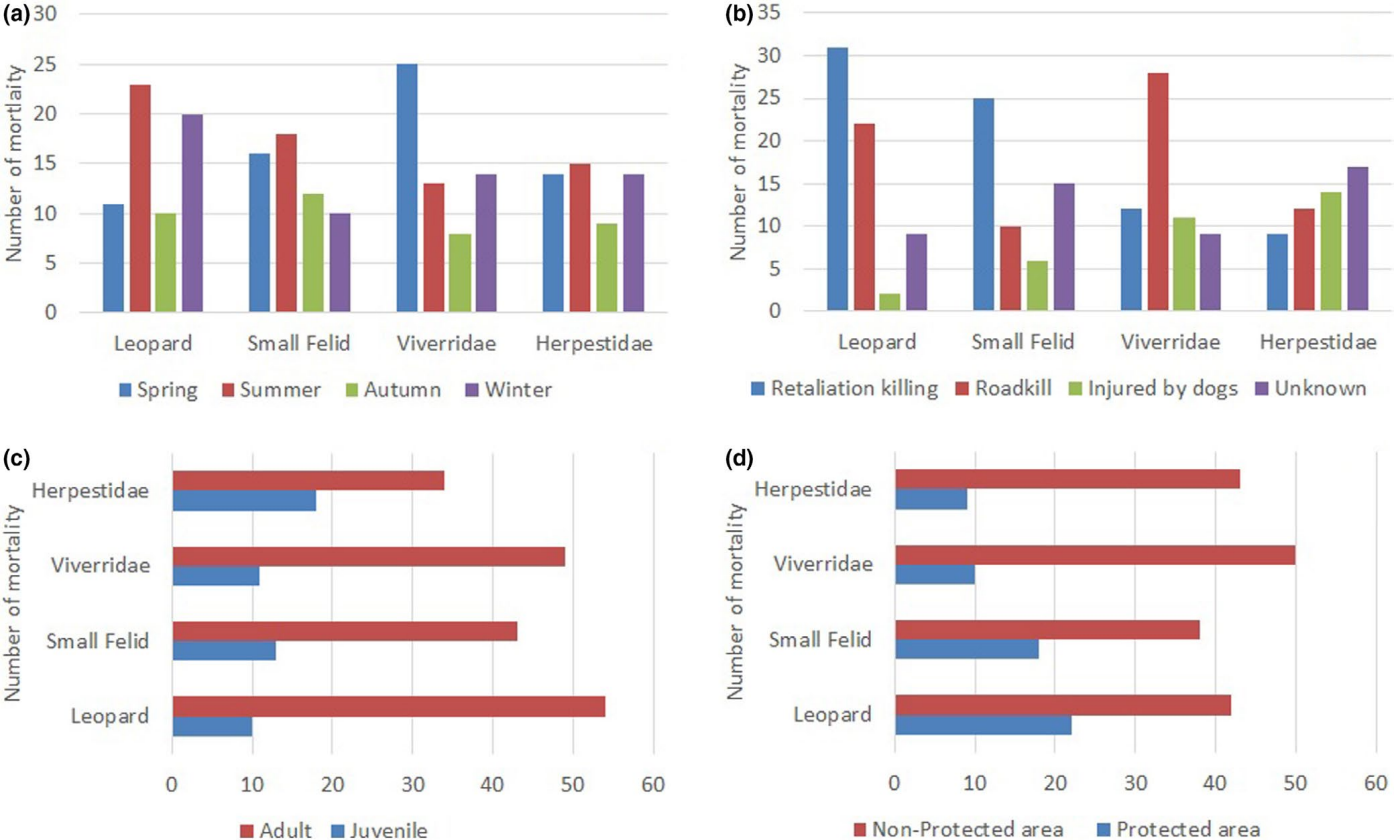
Bar graphs representing (a) number of mortality of four groups of carnivores according to seasons. (b) Number of mortality of four groups of carnivores according to cause. (c) Number of mortality of four groups of carnivores according to age category. (d) Number of mortality of four groups of carnivores according to protected area status

Among the total studied cases (*n* = 232), number of felid mortality was highest (51% out of total mortality cases), followed by Viverridae (25%) and Herpestidae (22%). Cases of retaliation killing were the most frequent cause of fatalities for felids (both leopard and small felids), whereas roadkill and injuries caused due to feral dogs were highest for Viverridae and Herpestidae, respectively (Figure 2). Chi‐squared test for goodness of fit was performed to examine the distribution of mortality of each taxonomic group with the causes. The test indicated that leopard mortality differed significantly among the causes (X2(3, *N* = 64) = 31.62, *p* < .001). Similarly small felid mortality (X2(3, *N* = 56) = 14.42, *p* < .01) and Viverridae mortality (X2(3, *N* = 60) = 15.33, *p* < .01) also differed significantly among the causes whereas the Herpestidae mortality did not differ significantly (X2(3, *N* = 52) = 2.61, *p* > .1). The results also indicated that the felid mortality was highest at summer whereas Viverridae mortality peaked at spring season (Figure 2). Chi‐squared for goodness of fit indicated that leopard mortality (X2(3, *N* = 64) = 7.87, *p* < .05) and Viverridae mortality (X2(3, *N* = 60) = 10.26, *p* < .05) differed significantly among the seasons whereas the small felid mortality (X2(3, *N* = 56) = 2.85, *p* > .1) and Herpestidae mortality (X2(3, *N* = 52) = 1.69, *p* > .1) did not differ significantly among the seasons.

Among the total carnivore mortality, 78% of cases included adults. Similarly, 75% of cases were reported in non‐protected areas (Figure 2). Chi‐squared test of independence indicated that the mortality in protected and non‐protected areas differed significantly among the studied group of carnivores (X2(3, *N* = 232) = 8.26, *p* < .05) whereas the mortality among the age‐group did not differ significantly among the studied group of carnivores (X2(3, *N* = 232) = 6.34, *p* > .05).

### 3.2 Total risk zone for carnivores

**FIGURE 3:**
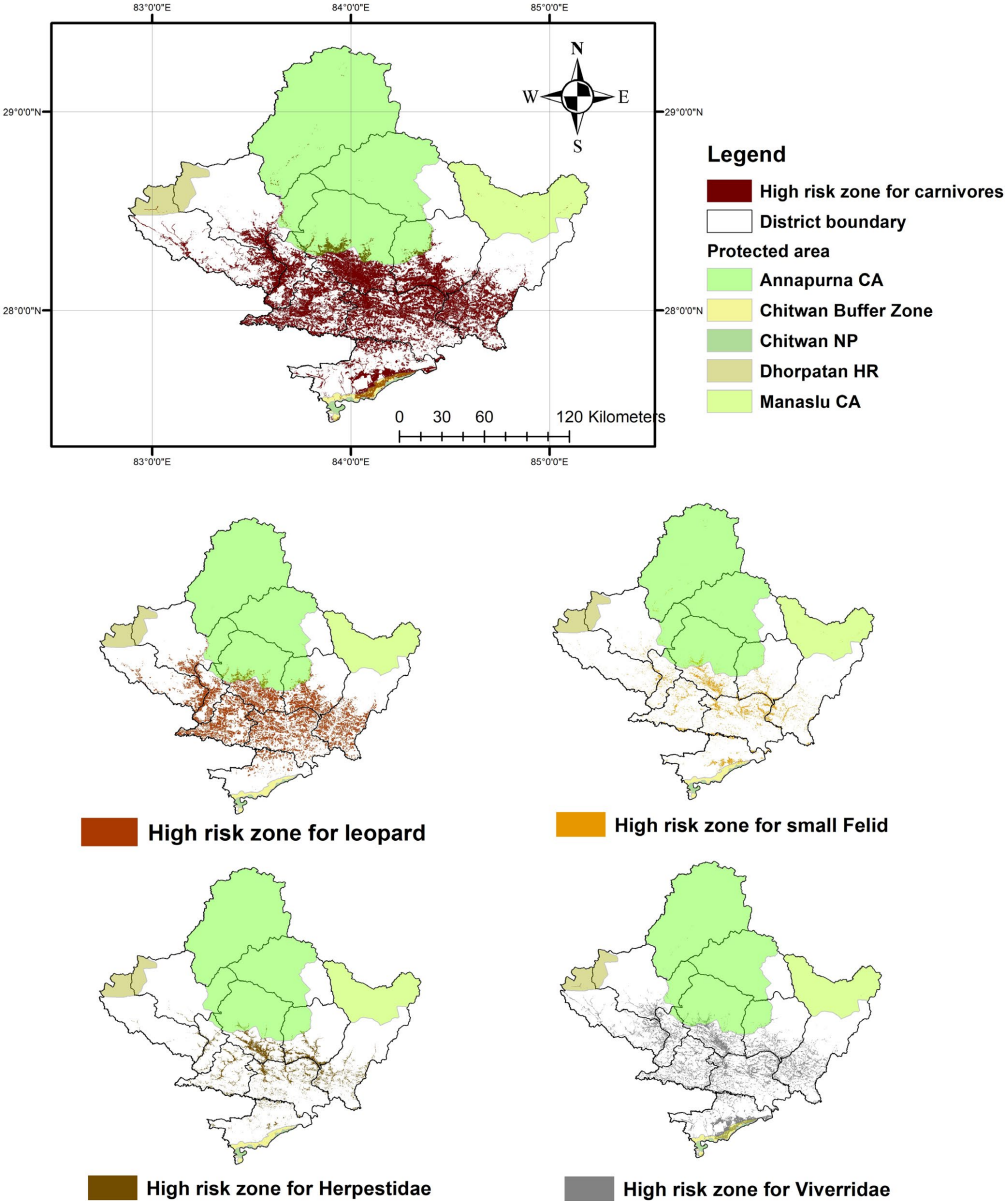
Map representing risk zone for carnivores inside the province. The risk zones of individual groups are not mutually exclusive and can overlap with each other. Overall risk zone was obtained from union of risk zone for four groups of carnivores

We found that a total of 3704 km^2^ (17% of total area of province) was potential high‐risk zone for carnivore mortality in the Gandaki province of Nepal (Figure 3). The Tanahun district demonstrated the highest risk followed by the Syangja, Gorkha, and Kaski districts, each representing over 10% of total risk area (Annex 2). Similarly, we found that the overall carnivore mortality was highest in the middle mountain range (85% of total risk area), whereas 229 km^2^ (6% of total risk area) was found inside protected areas. Additionally, agricultural lands were riskiest for carnivores, followed by forested areas, encompassing 65% and 28% of total risk area, respectively (Annex 2).

### 3.3 Risk zone for leopard and small felid

**FIGURE 4:**
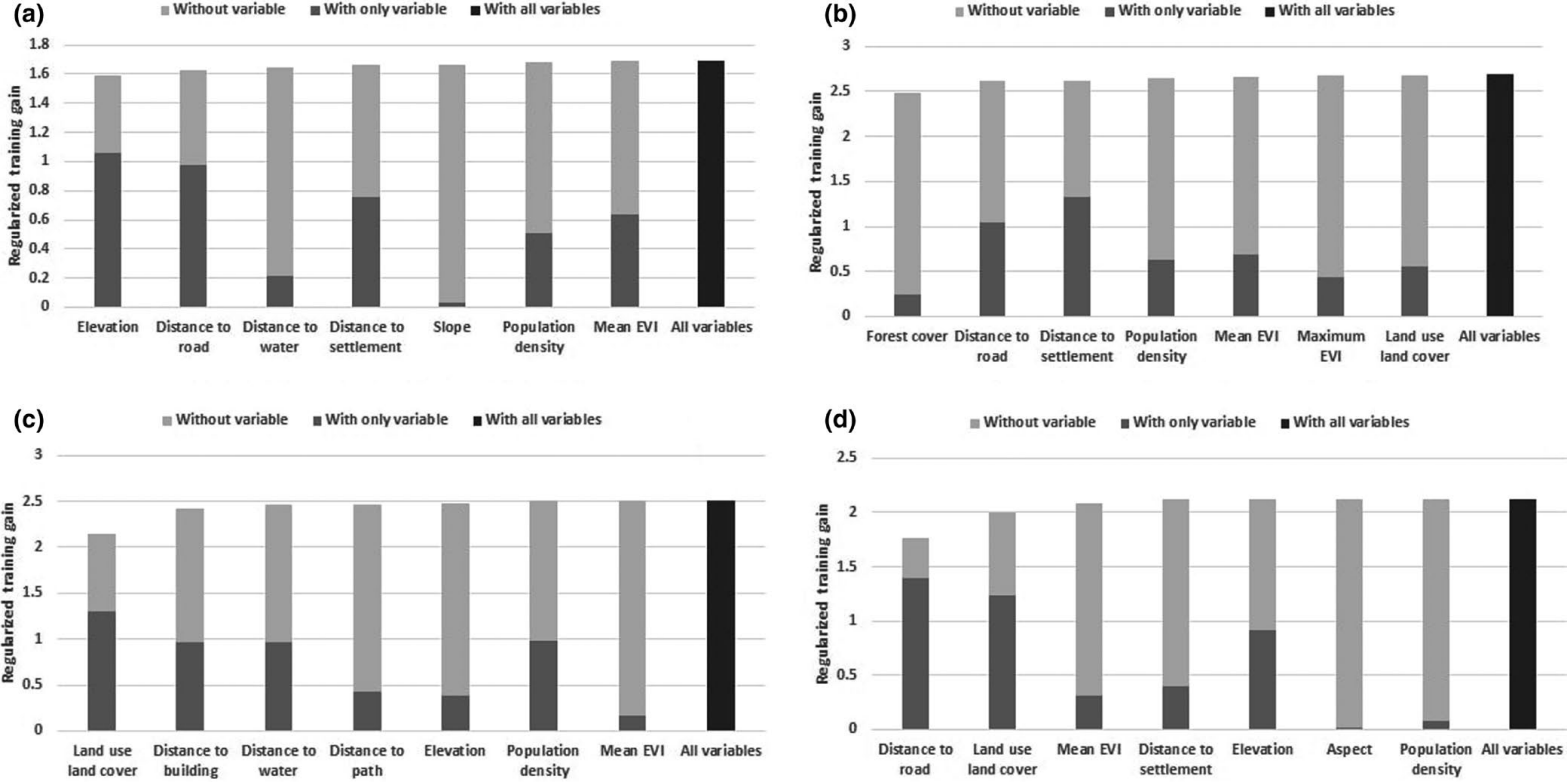
Importance of variables to train the model. Regularized training gain represents how much better the distribution fits the presence data compared with uniform distribution. “With only variable” represents result when only the particular variable is run, “Without variable” represents effect of removing particular variable from model and “With all variables” represent results of model when all variables are run. The figure shows jackknife regularized training gain of four carnivore groups (a) common leopard (b), small felid (c), Herpestidae, and (d) Viverridae

A total of 2322 km^2^ (11% of total area of province) was identified as potentially high risk for leopard casualties (Figure 3) (Annex 1). Out of the total risk‐prone areas for leopard inside the province, agricultural lands represented 54%, followed by forested land at 40%. The riskiest district for leopard casualties was Tanahun district (22% of total high‐risk area) followed by Syangja (17%), Kaski (15%), and Gorkha (15%) districts. The result of a jackknife test indicated that variables contributing most common leopard risk were elevation, distance to road, and distance to settlement (Figure 4). Similarly, a total of 660 km^2^ (3% of total area of province) inside the Gandaki province was concluded to be under high risk for small felid casualties, with the highest risk areas falling within agricultural lands (85%) (Figure 3) (Annex 1). The Tanahun and Kaski districts represented 26% and 20% out of total high‐risk area, respectively. Forest cover, distance to road, and distance to settlement were found to highly influence risk models (Figure 4).

### 3.4 Risk zones for Viverridae and Herpestidae

A total of 1589‐km^2^ area (7% of total area of province) was considered at high risk for Viverridae and 717 km^2^ (3% of total area of province) area at high risk for Herpestidae (Figure 3) (Annex 1). Again, agricultural areas represented the highest risk, 89% and 80% of total risk area for the two groups of carnivores, respectively. Tanahun (20%) and Nawalparasi (17%) established themselves the riskiest districts for Viverridae, whereas Tanahun (23%) and Kaski (23%) were found to be riskiest for Herpestidae. Land use/land cover and distance to building highly influenced Herpestidae risk zone, whereas Viverridae risk zone was influenced by distance to road and land use/land cover (Figure 4).

### 3.5 Accuracy of models

The average accuracy of the four models was good with AUC ≥ 0.89 and TSS ≥ 0.51. The AUC, TSS, and threshold used to convert the continuous probability map to binary high‐risk/low‐risk map of each models are represented in Table 3.

**TABLE 3:** Accuracy assessment for the Maxent risk zone models for four groups of carnivores. The table represents average AUC value, average TSS value and average threshold used to convert continuous probability map into binary high‐risk/low‐risk map. The values were obtained by averaging 10 model replicates for each group of carnivores

Group	AUC	TSS	Threshold
Large felid	0.89 ± 0.03	0.59 ± 0.08	0.211 ± 0.08
Small felid	0.97 ± 0.02	0.78 ± 0.07	0.147 ± 0.04
Viverridae	0.93 ± 0.08	0.53 ± 0.2	0.323 ± 0.11
Herpestidae	0.89 ± 0.4	0.51 ± 0.02	0.191 ± 0.08

### 3.6 Factors influencing number of casualty and risk area (km^2^) within districts

**FIGURE 5:**
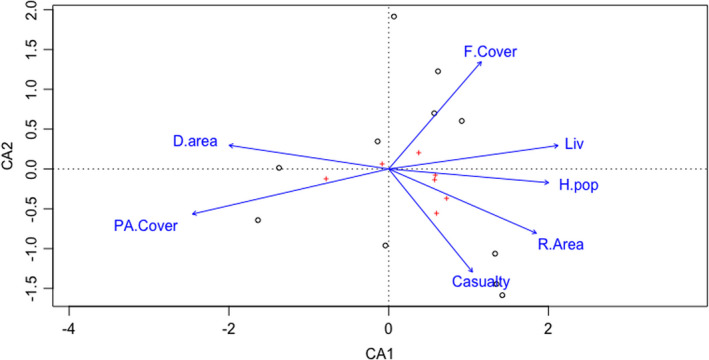
CAA ordination biplot representing the association between district wise variables (protected area cover, district area, forest cover area, number of livestock, human population, risk area km^2^, and number of casualty) within 11 districts

CCA indicated that number of casualty and high‐risk area (km^2^) within the districts were closely related (Figure 5). Both were positively associated with the human population whereas they revealed negative association with protected area cover within the districts. Monte Carlo 999 permutation test indicated that the analysis was significant (*p* < .05). CA1 primary axis variables explained 86.26% of variance (Table 4).

**TABLE 4:** Eigenvalues and their contribution to the scaled chi‐squared; the proportion of analysis explained by each canonical axes; and the cumulative proportion of importance they hold

Importance of components	CA1	CA2	CA3	CA4	CA5	CA6
Eigenvalue	0.2298	0.02489	0.007166	0.003628	0.0006955	0.0002233
Proportion explained	0.8626	0.09342	0.026894	0.013617	0.0026101	0.0008382
Cumulative proportion	0.8626	0.95604	0.982934	0.996552	0.9991618	1.0000000

## 4 DISCUSSION

Our results shed light to important component of carnivore conservation by providing insight on risk area and the attributes associated with anthropogenic mortality of carnivores. Prior studies also have conveyed the severity of threat that anthropogenic activities (retaliation killing, vehicle collision, attack from feral dogs) pose in maintaining carnivore populations, including studies such as anthropogenic mortality of black bear in the United States (Gantchoff et al., 2020), human‐induced mortality of leopard in Nepal (Thapa, 2014), risk of illegal poisoning of wild fauna in Spain (Mateo‐Tomas et al., 2012), the Iberian‐lynx road mortality in Spain (Garrote et al., 2018), the impact of human presence on the mortality of mammals in North America (Hill et al., 2020), and cause‐specific mortality of world's terrestrial vertebrates (Hill et al., 2019).

**FIGURE 6:**
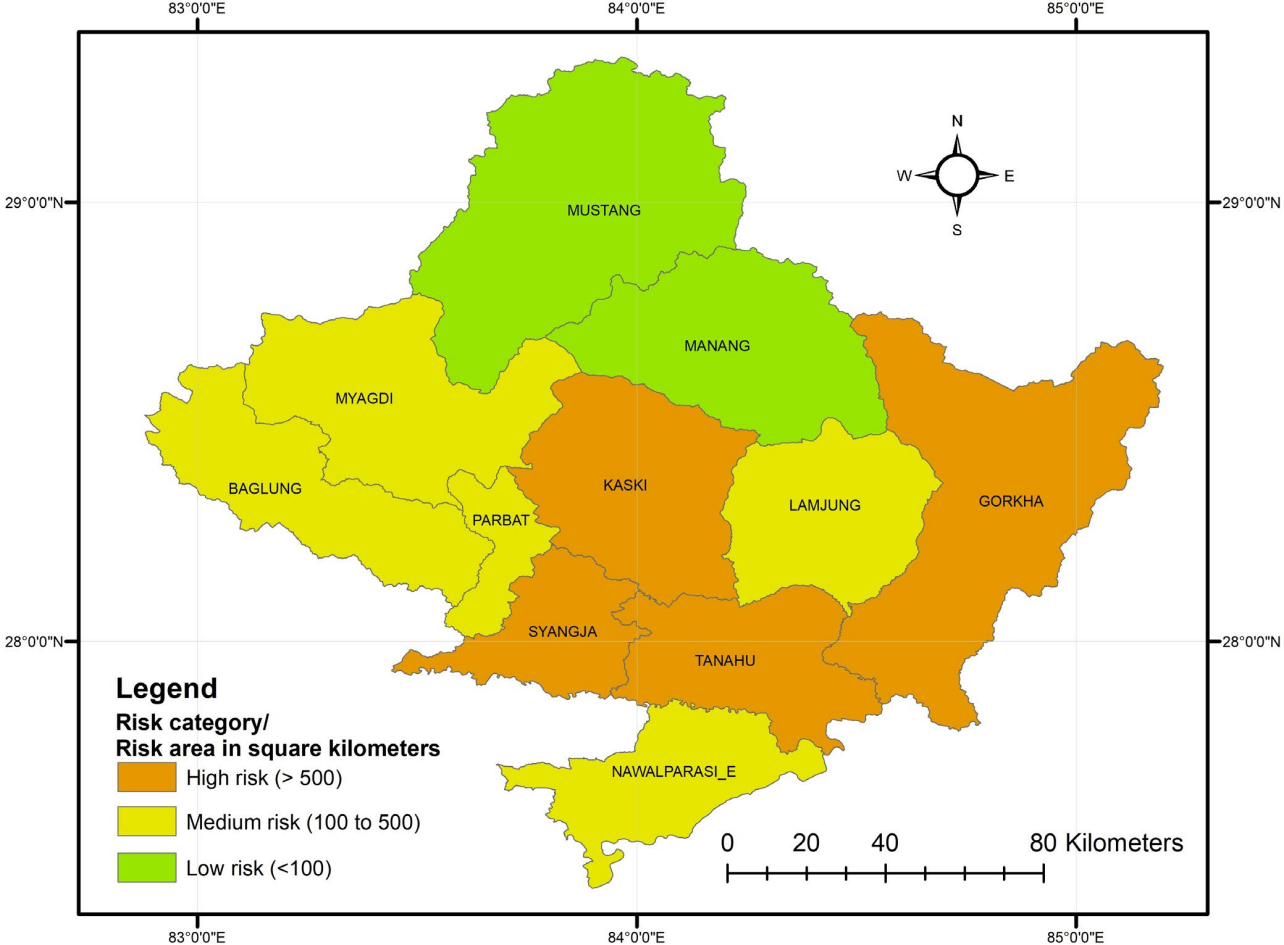
District wise carnivore risk zone categories where risk area greater than 500 km^2^ represents high‐risk district, risk area between 500 and 100 km^2^ represents medium risk district, and risk area less than 100km^2^ represents low‐risk district

A total of 3704 km^2^ (17% out of the total area of province) was identified as high risk for carnivore casualties. Districts within middle mountain regions of Gandaki province encompassed the highest risk area (Figure 6). In contrast, the lower risk zones were mostly located in the High Mountain and high Himalayan regions. Higher elevation areas were consistently at lower risk than low‐elevation areas, perhaps due to lower human population density and subsequently less anthropogenic pressure in high elevation areas. Despite this study identifying the Himalayan region as a low‐risk area, there have been reports of conflict in the region (Aryal et al., 2014; Pahari et al., 2021), which could easily have turned into wildlife casualty incidents. Low risk for carnivores in the Himalayan region could be explained by the presence of protected area management systems, which promote conservation of wildlife among local people (Schutgens et al., 2019), incentive‐based programs (Spiteri & Nepal, 2008), and compensation schemes in the event of livestock casualty, hereby preventing retaliatory killings. In contrast, the middle mountains encompassed the largest risk area, likely due to its large area and presence of densely populated cities with lower conservation incentives. The low‐ and moderate‐risk districts within the study area have lower human densities compared with high‐risk districts (UNFPA, 2017). High population densities are often associated with high anthropogenic pressure and hence pose a greater threat to local wildlife (Pietersen et al., 2014). The CCA performed in this study also represented positive association between human population and high‐risk zone (km^2^) for carnivore casualty (*p* < .05), supporting earlier findings. Anthropogenic mortality in mammals has been found to escalate with increasing human footprints and human‐associated impacts on the landscape (Gubbi et al., 2014, 2021; Hill et al., 2020). Results of our study also reinforce positive association between human population and number of carnivore casualty.

The importance of protected areas in preserving biodiversity and sustaining wildlife is well known (Acharya et al., 2016; Paudel & Heinen, 2015). Even though the protected areas cover 45% of the total area of the Gandaki province, only 6% of total risk area was included within protected areas. The CCA performed in this study also revealed negative association between protected area coverage and risk area (*r* = −0.35, *p* = < .05) for carnivores. This demonstrates the efficiency of protected area in reducing carnivore mortality. Protected areas inside Nepal are disproportionately located in high mountains and high Himalayan region (Paudel & Heinen, 2015). Subsequently, carnivores and other fauna of the middle mountains and lowlands are not adequately protected, with most forced to survive in or near human‐dominated forested landscapes (Paudel et al., 2012). Therefore, the main risk area (>94%) fell within unprotected areas. This result supports earlier findings of Acharya et al. (2016), who concluded that human‐dominated landscapes and the regions outside protected areas are the human–wildlife conflict hot spots in Nepal.

Larger mammals are likely to be at more risk due to increasing anthropogenic pressure (Hill et al., 2020; Thapa, 2014). Our study explored common leopard to be at more risk than other small carnivores. Since the common leopard is a large carnivore, it is the only species in our study which is capable of causing significant loss of livestock and has the potential to cause human casualties (Acharya et al., 2016, 2017). The mid‐hill districts represented the greatest risk areas for common leopard. Several incidents of HWC were recorded from these areas (Acharya et al., 2017; Bhandari et al., 2019). The incidences of human and livestock casualties have acted as a catalyst toward increasing leopard fatalities due to retaliatory killings. Agricultural lands inside the province represented the most at‐risk land‐use type for common leopard followed by forest areas and grasslands. The increasing urbanization and fragmentation of landscapes has caused significant reduction in the prey base of big cat species such as leopard (Puri et al., 2020; Schneider, 2001). Therefore, as highly adaptive and hardy species, leopards have shifted their preferences toward livestock as relatively easy prey (Abade et al., 2018; Shehzad et al., 2015). These agricultural lands, which harbor livestock such as cattle, buffalo, goats, and poultry, are increasingly being used by common leopards as hunting grounds (Kabir et al., 2014; Kshettry et al., 2017). Naha et al. (2020) reported high occurrence of conflict when livestock were allowed to graze freely within multi‐use areas. Forested areas were also established as quite risky for common leopard, due in part to the species’ need for large home ranges. Leopard often regularly patrol their range in search of a mate and to maintain their territory (Stein et al., 2011). Despite being elusive, this behavioral necessity of covering large home range makes the leopard more vulnerable to anthropogenic threats, hereby increasing casualty risk even within the forest and fringe areas (Acharya et al., 2017; Bhandari et al., 2019; Naha et al., 2020). Distances to roads and settlements were highly important variables to develop risk zone of common leopard. This result concurs within other documented literature (Edgaonkar & Chellam, 2002; Jacobson et al., 2016; Kumbhojkar et al., 2020) which mentioned high numbers of HWC events in areas of high anthropogenic pressure. Many studies have reported roadkill as one of the most significant causes of wildlife mortality (Baskaran & Boominathan, 2010; Sayyed & Mahabal, 2015).

In contrast to leopards, small felids were the group at lowest risk within the province. This may be due to their elusive nature and diet of mice and shrews, which are of low importance to humans. Small felids in the study (leopard cat and jungle cat) are sympatric species occurring in various ranges within the Indian subcontinent (Mukherjee et al., 2010). These two cats are considered being at similar risk, given their comparable size and overlapping prey preference (Majumder et al., 2011; Rajaratnam et al., 2007). Despite being at the lowest risk overall, this study predicted agricultural areas to represent the most risk for small felid mortality. Since agricultural areas harbor mice, rats, and shrew, they are presumed to attract small felids. In agricultural countries such as Nepal, most of the households in rural areas are built within close proximity of agricultural lands in order to make the fields and agricultural work more accessible and efficient. When these small felids wander through agricultural area in search for the prey species, they are bound to stumble upon humans, which may result in HWC (Inskip & Zimmermann, 2009). Hence, the vulnerability of small felids in the agricultural area is understandable.

Our study found that members of the Viverridae family were also at higher risk in agricultural lands. Civets are mostly omnivorous, but feed primarily on fruits and berries (Khan et al., 2019). These animals range agricultural lands in search of food and are consequently at risk from humans. Land use and distances to roads were recognized as the most important variables for prediction of Viverridae risk zones. Viverridae are nocturnal species, which forage at night (Bu et al., 2016), and as such, they are found upon road ways after dark, resulting in incidents of roadkill. Species within Herpestidae are more likely to occur in grassland and forestlands inside complex burrow systems (Mahmood & Nadeem, 2011). Distances to building and land‐use land cover were prominent risk variables for their mortality, indicating areas with high anthropogenic pressure. However, casualties of mongoose species may also be associated with their reputation as invasive and pest species (Ćirović et al., 2011; Hays & Conant, 2007).

This study incorporated important and representative groups of carnivores to develop risk zone models. The interaction between humans and carnivores results in complex intractable concerns which require proactive conservation approaches. The knowledge of risk zones and information on attributes of mortality are expected to address such complex issues by helping wildlife conservation authorities to be better prepared for swift and timely responses. The results from the current study are expected to aid in the development of specific conservation plans which addresses the severity of risks faced by carnivores, particularly the leopard. Additionally, the inclusion of protected areas in the middle mountain region is highly recommended to reduce future carnivore casualties. Further studies utilizing refined techniques of species distribution modeling alongside national‐level wildlife mortality database are recommended to explore the impact of anthropogenic, topographic, bio‐climatic, and other relevant environmental variables on the spatial–temporal pattern of wildlife mortality.

## CONFLICTS OF INTEREST

None declared.

## AUTHOR CONTRIBUTIONS

**Binaya Adhikari:** Conceptualization (lead); Data curation (lead); Formal analysis (lead); Investigation (equal); Methodology (equal); Writing – original draft (equal); Writing – review & editing (equal). **Kedar Baral:** Resources (equal); Supervision (equal); Validation (equal); Writing – review & editing (equal). **Shivish Bhandari:** Data curation (equal); Visualization (equal); Writing – original draft (equal); Writing – review & editing (equal). **Michelle Szydlowski:** Visualization (equal); Writing – review & editing (equal). **Ripu M. Kunwar:** Validation (equal); Writing – review & editing (equal). **Saroj Panthi:** Formal analysis (equal); Writing – review & editing (equal). **Bijaya Neupane:** Supervision (equal); Writing – review & editing (equal). **Raj Kumar Koirala:** Supervision (lead); Writing – review & editing (equal).

## Data Availability

The dataset that is associated with this study is available at dryad (https://doi.org/10.5061/dryad.wpzgmsbnk).

## ANNEX 1

Proportion (%) among the high‐risk area for carnivores according to district, physiographic zones, and protected areas within Gandaki province. Here, the total percentage represents total high‐risk area within the province

**Table:**

District	% out of total risk area	Land use	% out of total risk area
Tanahun	21.01	Agriculture area	65.15
Syangja	14.53	Forest	28.05
Gorkha	14.48	Grassland	2.10
Kaski	14.43	Built‐up area	1.42
Nawalparasi East	9.57	Shrubland	1.25
Lamjung	9.52	Barren area	1.10
Baglung	6.80	Water body	0.72
Parbat	6.25	Snow/glacier	0.01
Myagdi	3.20		
Mustang	0.19		
Manang	0.03		
Total	100% (3704 km^2^)	Total	100% (3704 km^2^)
Physiographic zones	% out of total risk area	Protected area	% out of total risk inside (PA)
Middle Mountain	85.10	Annapurna CA	61.93
High Mountain	7.54	Chitwan BZ	35.33
Siwalik	6.87	Dhorpatan HR	1.06
High Himalayas	0.43	Chitwan NP	0.93
Terai	0.05	Manaslu CA	0.75
Total	100% (3704 km^2^)	Total	100% (229 km^2^)

## ANNEX 2

Total high‐risk area (%) for each taxonomic group in various sub‐layers. The high‐risk area for each group of carnivore is not mutually exclusive and can overlap with each other. Each category represents % of total high‐risk area within the province whereas the protected area category represents % of total high‐risk area within the protected area in province

**Table:**

Category	Sub‐category	Risk area (%) for four groups of carnivores			
		Large felid	Small felid	Viverridae	Herpestidae
Land use/land cover	Forest	40%	6%	7%	10%
	Shrubland	2%	1%	1%	1%
	Grassland	2%	2%	1%	1%
	Agriculture area	54%	85%	89%	80%
	Barren area	1%	1%	0%	2%
	Water body	0%	1%	0%	3%
	Snow/glacier	0%	0%	0%	0%
	Built‐up area	1%	4%	1%	4%
	Total % and risk area	100% (2322 km^2^)	100% (660 km^2^)	100% (1589 km^2^)	100% (717 km^2^)
Districts	Baglung	6%	4%	9%	2%
	Gorkha	15%	13%	13%	16%
	Kaski	15%	20%	14%	23%
	Lamjung	11%	9%	7%	14%
	Manang	0%	0%	0%	0%
	Mustang	0%	1%	0%	0%
	Myagdi	3%	2%	3%	1%
	Nawalparasi East	3%	10%	17%	6%
	Parbat	7%	6%	6%	4%
	Syangja	17%	11%	12%	11%
	Tanahu	22%	26%	20%	23%
	Total % and risk area	100% (2322 km^2^)	100% (660 km^2^)	100% (1589 km^2^)	100% 717 km^2^)
Physiographic zones	High Himalayas	0%	1%	0%	0%
	High Mountain	7%	5%	8%	2%
	Middle Mountain	93%	88%	79%	96%
	Siwalik	1%	7%	14%	3%
	Terai, Madhesh	0%	0%	0%	0%
	Total % and risk area	100% (2322 km^2^)	100% (660 km^2^)	100% (1589 km^2^)	100% (717 km^2^)
Protected areas	Annapurna CA	100%	86%	33%	76%
	Chitwan BZ	0%	11%	63%	18%
	Chitwan NP	0%	0%	1%	5%
	Dhorpatan HR	0%	0%	2%	0%
	Manaslu CA	0%	4%	0%	0%
	Total % and risk area	100% (92 km^2^)	100% (38 km^2^)	100% (127 km^2^)	100% (17 km^2^)
